# Smoking, Mental Illness, and Public Health

**DOI:** 10.1146/annurev-publhealth-031816-044618

**Published:** 2016-12-16

**Authors:** Judith J. Prochaska, Smita Das, Kelly C. Young-Wolff

**Affiliations:** 1 Stanford Prevention Research Center, Department of Medicine, Stanford University, Stanford, California 94305; 2 Department of Psychiatry and Behavioral Sciences, Stanford University, Stanford, California 94305; 3 Division of Research, Kaiser Permanente Northern California, Oakland, California 94612

**Keywords:** smoking, tobacco, mental illness, psychiatric, depression, substance use

## Abstract

Tobacco use remains the leading preventable cause of death worldwide. In particular, people with mental illness are disproportionately affected with high smoking prevalence; they account for more than 200,000 of the 520,000 tobacco-attributable deaths in the United States annually and die on average 25 years prematurely. Our review aims to provide an update on smoking in the mentally ill. We review the determinants of tobacco use among smokers with mental illness, presented with regard to the public health HAVE framework of “the host” (e.g., tobacco user characteristics), the “agent” (e.g., nicotine product characteristics), the “vector” (e.g., tobacco industry), and the “environment” (e.g., smoking policies). Furthermore, we identify the significant health harms incurred and opportunities for prevention and intervention within a health care systems and larger health policy perspective. A comprehensive effort is warranted to achieve equity toward the 2025 Healthy People goal of reducing US adult tobacco use to 12%, with attention to all subgroups, including smokers with mental illness.

## OVERVIEW AND SCOPE

This review covers the prevalence, consequences, and correlates of tobacco use among persons with mental illness. With an emphasis on public health, we apply the HAVE model (host–agent–vector–environment) to consider cell-to-society factors implicated in the disconcertingly high levels of tobacco use in this group and the consequential tobacco-related disparities evident in increased morbidity and mortality. Although initially designed for application to the study of infectious diseases, the HAVE model has also been applied effectively to tobacco control (46, 86).

This review is timely because smoking among individuals with mental illness has gained attention, and research in this area has grown exponentially. A recent bibliometric analysis of the literature on tobacco and mental illness documented a steady increase in research outputs from the two-year periods of 1993–1995 (*n* = 65) to 2003–2005 (*n* = 153) to 2013–2015 (*n* = 329) (81). Notably, the study designs remained predominantly descriptive in form (>80%), with few experimental studies testing cessation interventions (<13%).

With an emphasis on informing next steps for solutions, we also review the evidence and identify opportunities for intervention with consideration of health care systems and public policies. Our perspective here is public health oriented, so we refer those seeking a more clinical focus to prior reviews (54, 89). With attention to emerging nicotine delivery systems, we include research on electronic cigarette and vape products, which have flooded the market with evidence of uptake and use among smokers with mental health concerns (57, 94). We close with consideration of changes in US health care policies to afford and avail preventive care, including tobacco treatment, to the broad population of smokers, including persons with mental illness.

## THE HAVE MODEL: HOST–AGENT–VECTOR–ENVIRONMENT

Applied to tobacco initially by Orleans & Slade (86) in 1993 and further considered by Giovino et al. (46) in 2002, the HAVE model, as a public health framework, considers the diversity of influences on the prevention and control of tobacco use: The four broad domains are as follows:

- Host refers to tobacco user characteristics (e.g., biobehavioral, social/cognitive, mental health);
- Agent represents the tobacco product characteristics (e.g., nicotine content, delivery, flavorings);
- Vector indicates tobacco industry efforts (e.g., research, development, advertising, distribution); and
- Environment represents broader community and policy structures (e.g., taxation, smoking bans, insurance coverage, retailers).

While traditionally used to characterize infectious disease, the multidimensional HAVE model is well suited for considering the complex nature of influences determining tobacco use among smokers with mental illness.

## HOST: TOBACCO USERS

Host refers to the user of the tobacco product, our point of interest being individuals with mental illness. Within the scope, we consider any diagnosable psychiatric disorder such as depression, schizophrenia, and anxiety disorders and include non-nicotine substance use disorders, which have a similarly high co-occurrence with tobacco use with shared determinants and high co-occurrence with other forms of mental illness.

### Prevalence of Smoking

Reported in the United States, the United Kingdom, and Australia, smoking is two to three times more prevalent among people with mental illness, when compared with the general population (38, 75, 78). When assessed by psychiatric diagnoses, smoking prevalence is particularly high (almost fivefold greater) among those with schizophrenia, bipolar disorder, post-traumatic stress disorder (PTSD), and alcohol/illicit drug use disorders. Notably, the association is stronger with PTSD diagnosis than with trauma exposure alone (43). Depression is twice as common in smokers than nonsmokers, and four times as common in heavy smokers (66). Smoking prevalence increases with a greater number of mental disorders, ranging from 18% for people with no mental illness to 61% for people diagnosed with 3 or more mental disorders (40).

The elevated prevalence of smoking among individuals with mental illness is due in part to disparities in tobacco use reductions over time. In the mid-1960s, about one-half of adult men and one-third of adult women in the United States smoked cigarettes (133). Owing to recognition of the health harms of smoking and secondhand smoke, availability of cessation treatments in general medical settings, and the expansion of smoke-free air laws in workplaces and public areas, tobacco use among US adults nationally has declined steadily over the past 6 decades to 19% of men and 15% of women (62). Similar reductions in smoking have not been observed among individuals with mental illness (28, 34, 145). The 2025 Healthy People goal is to decrease US adult tobacco use to 12%, which would be a substantial (50% or greater) reduction as a target for smokers with mental illness (134). People with mental illness who are socioeconomically disadvantaged are even more at risk of smoking: 48% of people with mental illness who live below the poverty level smoke, compared with 33% of those with mental illness who live above the poverty level (29).

Mental illness is associated with heavier smoking, greater nicotine dependence, greater withdrawal symptoms when quitting, and lower quit rates (19, 45, 70, 78, 90, 124). With greater, heavier, and more chronic use, research estimates that individuals with mental illness consume nearly half of cigarettes sold in the United States (51, 70), with similar estimates of consumption in the United Kingdom, Australia, and New Zealand. Despite their high levels of tobacco use and the potential challenges of living with mental illness, this population’s motivation to quit smoking is high and comparable with estimates of intention to quit smoking in the general population (2, 83, 91, 98). Among smokers hospitalized with mental illness, 65% were interested in quitting tobacco use (114).

### Self-Medication versus Causation or Bidirectional Models

The self-medication hypothesis posits that individuals with mental illness smoke to lessen their symptoms (11). Tobacco companies funded research in support of this hypothesis (96). In contrast, numerous studies indicate that smoking may cause depression, anxiety disorders, and schizophrenia and is a gateway to problematic substance use (20, 50, 64, 121, 138). Evidence also shows that secondhand smoke exposure is related to the development of depression, generalized anxiety disorders, attention-deficit/hyperactivity disorder, and conduct disorder (13, 15). Bidirectional models maintain that smoking and psychiatric symptoms influence each other (139).

In schizophrenia, smokers experience increased psychiatric symptoms and more hospitalizations compared with nonsmokers. Heavier smokers have increased positive symptoms (hallucinations, delusions) and reduced negative symptoms (anhedonia, alogia, flat affect) compared with nonsmokers and nondaily or light smokers (148). Cigarette smoking induces the metabolism of some psychiatric medications leading to lower therapeutic blood levels and the need for higher doses (146). A number of studies, in youth and adults, cross-sectional and prospective, have found that current smoking is predictive of future suicidal behavior, independent of depressive symptoms, prior suicidal acts, and other substance use (21, 85, 87). Furthermore, longer lifetime smoking (>40 years versus ≤10 years) is associated with higher odds of suicide [odds ratio (OR) = 2.26, 95% confidence interval (CI) (1.30, 3.93)] (12). Notably, quitting smoking appears to mitigate the risk (32).

The self-medication hypothesis—that smokers need to smoke to manage their mental health symptoms—drove concerns that treating smoking would worsen depression, anxiety, psychosis, and other substance use. These beliefs combined with perceptions that tobacco use is a chronic, rather than acute, concern have been significant barriers to treating tobacco use in mental health settings (79). Newer research, however, indicates that quitting smoking is associated with improvements in mental health, including reductions in depression, anxiety, and PTSD symptoms (63, 80, 88). In a study of veterans quitting smoking, tobacco abstinence at follow-up was associated with mental health functional improvement, including depression, psychotic symptoms, and emotional lability (69). A meta-analysis of 26 tobacco intervention studies found that smoking cessation was significantly associated with decreased anxiety, depression, and stress and improvements in overall mood and quality of life (125). Notably, the strength of this relationship was invariant based on presence/absence of a psychiatric diagnosis. Another meta-analysis, focused on smokers in treatment for substance use disorders, found that tobacco-cessation interventions were associated with a 25% increased likelihood of sobriety from alcohol and drugs relative to usual care (90). In a randomized trial with smokers recruited from inpatient psychiatry, the tobacco-cessation intervention was associated with a significantly lower likelihood of rehospitalization (95). In contrast with clinical lore that treating smoking would harm sobriety or mental health recovery, the research indicates enhanced clinical outcomes associated with tobacco-cessation treatments.

### Tobacco-Related Morbidity and Mortality

Smoking remains the leading cause of preventable death globally, responsible for more than a half a million deaths annually in the United States, about one of every five deaths (27) (see the sidebar titled Tobacco Use and Mental Illness: A Compounded Problem). Tobacco use kills nearly one in two long-term users, with one-third of those deaths related to cardiovascular disease and stroke, another one-third being cancer related, and about one-fifth due to respiratory diseases (133). Pervasively harmful, smoking affects almost every bodily organ. Contrary to earlier beliefs that persons with mental illness and their relatives may harbor protection from tobacco’s harms (55, 102), researchers now recognize that those with mental illness are disproportionately affected by smoking-related morbidity and mortality.

**TOBACCO USE AND MENTAL ILLNESS: A COMPOUNDED PROBLEM**

Tobacco remains the leading preventable cause of death globally, affecting people with mental illness and substance use disorders disproportionately. Propagating this matter are the tobacco industry’s extensive efforts in research, marketing, and advertising; product development and delivery; biophysiologic processes of nicotine addiction; and historical features of mental health and addiction treatment settings with a general failure to treat tobacco. Evidence-based efforts for reducing the major public health harms and burden of tobacco include individual treatments, technologic innovations, provider training and reimbursement, and broader policy measures.

In the mid-1950s, tobacco companies noted the apparent low incidence of lung cancer among patients with schizophrenia, a group known to smoke heavily. With interest, they questioned the feasibility of quantifying this association (77). If the finding could be substantiated, perhaps it would allay concerns of smoking causing lung cancer. The reality, at the time, was that smokers with schizophrenia were not living long enough to get cancer; instead, they were dying from tuberculosis and syphilis and institutionalized in settings less likely to detect their cancer.

Research has now clearly demonstrated that people with mental illness carry a disproportionate share of medical burden from tobacco use (35, 60, 76, 108, 117). In the United States, smokers with mental illness account for more than 200,000 of the 520,000 tobacco-attributable deaths annually and are dying on average 25 years prematurely, with leading causes being chronic disease, most of which are tobacco related (33). Comparable estimates in years of life lost have been reported in Australia, New Zealand, and Canada (74). A recent epidemiologic study in California found that approximately half of the deaths in those who had been hospitalized for schizophrenia, bipolar disorder, or major depressive disorder were due to diseases identified as causally linked to tobacco use (26). A similar analysis with a focus on deaths among those hospitalized for opioid-related conditions concluded that the major causes of death were related to tobacco and alcohol, not to opioids (135). Smoking has synergistic negative health effects with other substances, with a 38-fold greater risk of developing cancers of the mouth and throat among those who abuse both alcohol and tobacco (131).

In addition to adverse health effects, there are many other negative consequences of smoking in those with mental illness. On an economic level, smoking can affect treatment and survival by consuming funds and effort to obtain cigarettes; a study of smokers with schizophrenia estimated that median spending on cigarettes was 27% of monthly incomes (120). On a social level, smokers experience discrimination and stigma (22, 111, 123), contributing to increased alienation and poorer mental health.

## AGENT: CIGARETTES AND EMERGING NICOTINE PRODUCTS

While cigarettes are the most common nicotine product, electronic cigarettes are gaining popularity.

### Cigarettes

A cigarette is a cylinder of tobacco rolled in paper for smoking. Nicotine, a naturally occurring pesticide on the tobacco leaf, is the primary psychoactive and addictive chemical in cigarettes. Added to the shredded tobacco mixtures are humectants to keep the tobacco moist (e.g., propylene glycol) and flavoring products (e.g., menthol), flavor enhancers, sugars, and chemicals, such as ammonia, which contribute to the addictive properties of cigarettes, especially when burned. People with mental illness are more likely than those in the general population to smoke cigarettes, menthol cigarettes, and emerging nicotine products.

### Menthol

Starting in the 1930s, menthol (a substance naturally found in mint plants) was added to many cigarettes after it was accidentally found to provide cooling and anesthetic properties to the smoke, making it easier to inhale (99). In the United States, approximately 27% of all cigarettes sold nationally are characterized as menthol cigarettes (128). Menthol cigarettes have been aggressively marketed as healthier and safer alternatives to regular cigarettes to groups at high risk for smoking, including African Americans, Native Hawaiians, adolescents, and low-income communities (44, 47, 104, 141). An authoritative 2011 report to the US Food and Drug Administration (FDA), however, concluded that there is sufficient scientific evidence to show that menthol cigarettes promote experimentation, regular smoking, and increased likelihood of addiction in youth smokers and are associated with less success with quitting among African American smokers (128).

Several studies have now documented a greater proportion of menthol use among smokers with mental illness. Analysis of Florida state data indicated that smokers with poorer mental health were more likely to smoke menthol than nonmenthol cigarettes (137), and in a national sample, menthol use was more likely among smokers with severe psychological distress than among smokers with none or mild distress (58). Furthermore, in a sample of 1,042 adult smokers hospitalized in the San Francisco Bay Area with serious mental illness, menthol use was twofold greater (57%) relative to the general population of smokers in California (24%) (143). A follow-up study with 481 smokers hospitalized with mental illness examined tobacco preference in relation to price sensitivity and sensory correlates and found menthol-only users were the most flavor-loyal (144).

### Electronic Nicotine Delivery Systems

Cigarettes represent about 90% of tobacco/nicotine use, although the market for electronic nicotine delivery systems (ENDS; e.g., e-cigarettes, e-hookahs, vape pens) is growing in the United States and internationally (3, 10, 39, 49, 65). Notably, the tobacco industry controls more than 80% of the e-cigarette US market in unit sales (52). ENDS are battery-powered devices that generate an aerosol, typically containing nicotine, for inhalation. Vigorous debate in the public sphere and scientific literature concerns the potential for ENDS as a safer alternative to tobacco cigarettes for smokers unable or unwilling to quit or for use as a cessation aid (31, 41). Proponents argue that ENDS are appealing to smokers as a harm-reduction tool because they mimic cigarettes in appearance, method of inhalation, production of smoke-like aerosol, and taste and are likely safer than tobacco cigarettes. In terms of exposure risks, analysis of 12 first-generation (cigarette-like) products of ENDS found varying levels of toxic and carcinogenic compounds in the aerosols, about 9–450 times lower than cigarette smoke, and toxicants in some brands, on some measures, were comparable with those in the nicotine replacement therapy (NRT) inhaler (48). Research on ENDS is limited but growing; most studies to date have been descriptive. Among smokers with mental illness, research shows a similar rise over time in ENDS ever use (94). Among 188 veterans who were current smokers of tobacco cigarettes and who were seeking mental health and/or substance use services, 31% reported current e-cigarette use (57). Notably, dual use was high, and few reported that e-cigarettes helped them reduce or quit cigarette smoking. Additional data are needed to more fully understand the long-term potential of these products for harm/harm reduction, particularly in vulnerable groups of smokers, including those with mental illness.

## VECTOR: INDUSTRY EFFORTS

Using both direct (i.e., distribution, advertising, funded research, scientific publications, meetings) and indirect (i.e., policy effort) strategies, such as opposition to hospital smoking bans in psychiatric units/settings (96), the tobacco industry has promoted smoking in psychiatric patients.

### Tobacco Industry Marketing

Many tobacco print advertisements incorporate imagery of joyful smokers, who are engaged socially, with freedom from stress and anxieties. Fewer, though with some notable exceptions, have connected to mental illness more patently. For example, an advertisement for Merit cigarettes headlined the word “Schizophrenic” asserting that “for New Merit having two sides is just normal behavior” (96). Another campaign, RJ Reynolds’s Project SCUM (SubCulture Urban Marketing) targeted San Francisco’s alternative lifestyles with attempts to cobrand cigarettes with other drug use by saturating “head shops” (101).

### Tobacco Industry Research and Collaborators

The tobacco industry monitored the scientific literature and funded internal and external research on the self-medication hypothesis, described above. A search of documents in the Truth Tobacco Industry Library identified 28 proposals relating to schizophrenia, of which 7 were ultimately funded, all seeking to expose hypothesized self-medicating effects of nicotine and smoking for those with schizophrenia (96). One proposal titled “Tobacco Smoking as a Coping Mechanism in Psychiatric Patients” asserted that “[i]f tobacco can be shown to be an efficient form of self-medication for these patients then this would be [a] significant bonus for the tobacco industry” (67). The 21 unfunded proposals largely concerned the study of the high smoking prevalence, varied health harms (e.g., cancers, medication interactions), and nicotine withdrawal effects.

Tobacco industry–funded researchers published review articles, hosted scientific meetings, and edited textbooks concerning the merits of nicotine and nicotine analogs in the treatment of schizophrenia. One example titled, “Nicotine: Helping Those Who Help Themselves?” (107), even recommended nicotine use among nonsmokers with schizophrenia. The tobacco industry also enlisted psychiatrists as expert witnesses in court cases and to testify before congress and the FDA, a common thread being the assertion that nicotine is not addictive (61). Furthermore, some have characterized nicotine withdrawal and difficulty with quitting as more to do with tobacco users’ anxiety and personality disorders than with the drug effects of nicotine.

### Tobacco Retailers

The tobacco retail environment has been identified as a potential vector contributing to tobacco-related disparities among individuals with mental illness. A study of 1,061 smokers with serious mental illness residing in the San Francisco Bay Area linked participants’ geocoded addresses with tobacco retailer licensing data and reported the sample median of 3 retailers within 500 m and 12 within 1 km of participants’ residences, which was twofold greater than for the general Bay Area population (142). Furthermore, tobacco retailer density was associated with poorer mental health, greater nicotine dependence, and lower self-efficacy for quitting smoking. The study suggested that smokers with mental illness, in particular, may benefit from progressive environmental protections that restrict tobacco retail licenses and reduce aggressive point-of-sale marketing.

## ENVIRONMENT: MENTAL HEALTH SETTINGS AND SMOKE-FREE POLICIES

Environmental factors propagate the use of tobacco among those with mental illness. Unfortunately, historically, mental health care settings and providers have contributed to the problem.

### Provider Behavior

Despite the increased mortality, morbidity, and co-occurrence of smoking with mental illness, treatment has been limited. In a survey by the American Association of Medical Colleges of more than 3,000 physicians, psychiatry was the specialty least likely to address tobacco with patients: only 23% provided assistance with quitting smoking and fewer (11%) provided treatment referrals (1). Nearly half (47%) of surveyed psychiatrists felt patients had more immediate problems to address, and 22% reported that cessation heightens other symptoms (attitudes more common among psychiatrists than all other specialties surveyed). A recent meta-analysis of 38 studies with 16,369 mental health professionals concluded that 42% [95% CI (36, 49)] perceived barriers to smoking cessation intervention, 41% [95% CI (30, 51)] had negative attitudes toward smoking cessation, and 45% [95% CI (32, 58)] had permissive attitudes toward smoking (113). The most commonly held beliefs were that patients with mental illness are disinterested in quitting [51%, 95% CI (33, 69)] and that quitting smoking is too stressful for these patients [38%, 95% CI (16, 63)]. In an online patient survey of 519 smokers with bipolar disorder, few reported that a psychiatrist (27%), therapist (18%), or case manager (6%) had ever advised them to quit smoking, and several reported discouragement to quit from mental health providers (97). Multiple factors likely contribute to this problem, including the lack of mental health professional education in treating tobacco use and a long history of tobacco use in mental health treatment settings (discussed next). For example, a review of 26 studies on smoking bans in psychiatric facilities found that staff believed that cigarettes were important for self-medication and that smoking bans would worsen patients’ mental health symptoms and increase behavioral problems, though research has proven otherwise (72).

### Mental Health Settings

Cigarettes have a history of promoted use in mental health settings. Until recently, clinicians in psychiatric treatment centers would smoke with patients, provide cigarettes as incentives, or give cigarettes in response to agitation (73, 140). Documents in the Truth Tobacco Industry Library (http://legacy.library.ucsf.edu) show that the tobacco industry was supplying either free, low-cost, or tax-free cigarettes to psychiatric institutions (96), with requests as recent as 2000. In a survey study conducted in the United Kingdom in 2003 with inpatient psychiatry nursing staff, 53% believed clinicians smoking with patients was therapeutic, and 22% believed that cigarettes should be handed out to patients as part of therapy (122).

In 1993, the Joint Commission banned smoking in hospitals across the United States. The Joint Commission’s efforts to include psychiatric units and hospitals in the smoke-free policy were thwarted by mental health advocacy groups, in communication with tobacco companies, citing smoking as a patient’s rights issue (96). This effort was greatly unfortunate, given the aforementioned burden of tobacco on these populations, the clear harms of secondhand smoke exposure (contributing to 40,000 deaths per year nationally), and the high potential for tobacco smoke to serve as a sensory cue trigger for craving and relapse. In contrast, alcohol is not permitted on addiction treatment and mental health campuses in recognition of the need for a culture of recovery protected from obvious relapse triggers (147).

While smoking bans are still not federally mandated, the number of smoke-free state-run psychiatric hospitals continues to rise in the United States from 20% in 2005 to 79% in 2011 (109). In Europe, complete smoking bans in inpatient psychiatry are still debated; patients are often permitted to smoke outside (9). In the United States, a 2013 front-page article in the *New York Times* on smoking bans in psychiatric hospitals generated online public commentary revealing stigma and devaluation of life for persons with serious mental illness (23). Even when acute psychiatric settings ban smoking, the hospital stays are typically short (one week on average), and many patients are discharged to residential treatment settings where smoking is widely permitted or they return home to neighborhoods with a high density of tobacco retailers (142).

## TOBACCO-CESSATION TREATMENT STRATEGIES

While there is a growing appreciation and prioritization among tobacco researchers of the high smoking prevalence among those with mental illness (28, 34, 62), the lack of decline in use over time, despite population trends (29, 62, 134, 145), suggests that efforts at the general population level are not effectively addressing tobacco-related disparities in this group and may in effect be maintaining them (19, 70). Research is greatly needed to determine effective intervention strategies to reduce smoking and its burden for this disadvantaged group (62). Here, we review the evidence available to date and highlight several research gaps. Included are individual strategies (seven FDA-approved cessation medications, therapy/counseling) and broader policy strategies (smoking bans, health care coverage). When we refer to abstinence, it is with regard to abstinence from tobacco products.

### Individual Focused

#### Cessation pharmacotherapy

NRT, available in formulations of transdermal patch, gum, lozenge, inhaler, and nasal spray, provides nicotine to treat withdrawal and address physical dependence without exposure to toxic combustion products. All NRT formulations provide lower and slower-rising plasma nicotine concentrations compared with cigarettes, reducing the behaviorally reinforcing effects of smoking. Among studies in the general population, the different forms of NRT have comparable efficacy (53). In the emergency room setting, treating nicotine withdrawal with NRT (versus placebo) among smokers with schizophrenia increased cooperation and decreased agitation acutely (5). NRT has been available since 1984, yet a recent meta-analysis failed to identify a single randomized trial reporting six-month or greater abstinence outcomes of NRT monotherapy in the serious mentally ill versus placebo or other monotherapies (105); this is a major gap in the literature. For those with substance use disorders, a recent meta-analysis found that nicotine patches improved continuous abstinence at 6 months, and nicotine gum improved continuous abstinence at 12 months (127).

Bupropion, a blocker of dopamine and, to a lesser extent, norepinephrine reuptake, also has some nicotine receptor–blocking activity (115). A Cochrane review of seven randomized, placebo-controlled trials found that smokers with stably treated schizophrenia who used bupropion to aid smoking cessation were nearly three times as likely as those on placebo to be abstinent at the end of the drug therapy (129), with no worsening of schizophrenia or depressive symptoms.

Varenicline is a partial agonist of the *α*4β2 neuronal nicotinic acetylcholine receptor, which mediates dopamine release and is thought to be the major receptor involved in nicotine addiction. As a partial agonist, varenicline stimulates low-level agonist activity while competitively inhibiting binding of nicotine, reducing symptoms of nicotine withdrawal as well as reducing the reinforcement/reward associated with smoking. The FDA approved varenicline in 2006. Routine postmarketing safety reports revealed that an unknown proportion of patients treated with varenicline showed signs of neuropsychiatric symptoms. An FDA analysis of postmarketing reports for all cessation medications led to a box warning for both bupropion and varenicline in 2009, which showed more neuropsychiatric adverse reports relative to NRT.

With demonstrated efficacy, research shifted to examine the neuropsychiatric safety of bupropion and varenicline in randomized controlled trials where the denominator would be known. A 2015 meta-analysis of 39 randomized placebo-controlled trials of varenicline in the general population (*n* = 10,761) found no evidence of an increased risk of suicide or attempted suicide, suicidal ideation, depression, or death (126). A more focused meta-analysis of randomized placebo-controlled trials of studies with patients with severe mental illness found that both bupropion and varenicline were tolerable from a safety standpoint and more effective than placebo [OR = 4.51, 95% CI (1.45, 14.04) and OR = 5.17, 95% CI (1.78, 15.06), respectively] and were not significantly different from each other (105).

Published in 2016, the EAGLES (Evaluating Adverse Events in a Global Smoking Cessation Study) trial was a postauthorization safety study that was developed with the FDA, conducted in 16 countries, and designed to evaluate the neuropsychiatric safety of varenicline and bupropion versus placebo and nicotine patch in patients with and without a history of or current psychiatric disorder (7). In this study of 8,144 participants (4,116 psychiatric patients and 4,028 nonpsychiatric patients), moderate to severe neuropsychiatric effects were found at rates of 1.3% (varenicline), 2.2% (bupropion), 2.5% (nicotine patch), and 2.4% (placebo) in the nonpsychiatric cohort. In the psychiatric cohort, rates of moderate to severe neuropsychiatric effects were 6.5% (varenicline), 6.7% (bupropion), 5.2% (nicotine patch), and 4.9% (placebo). Compared with NRT and placebo, there was no significant risk difference for varenicline or bupropion overall or by cohort. The most common adverse event with varenicline was nausea (25%), and the most common adverse event with bupropion was insomnia (12%). In response to the findings from the EAGLES trial, in December 2016, the FDA announced removal of the box warning on varenicline.

Taken together, available evidence indicates that FDA-approved cessation pharmacotherapies have the potential to reduce the behaviorally reinforcing effects of smoking, to treat withdrawal, and to address physical dependence without exposure to toxic combustion for adults with mental illness. However, additional randomized trials on the efficacy and tolerability of NRT monotherapy in the serious mentally ill versus placebo or other monotherapies are critically needed.

#### Cessation counseling

Cessation counseling provided by a trained physician or therapist typically teaches behavioral techniques with support to address the ingrained habit of smoking. Group therapy offers the added value of fostering peer support and is likely to be more cost-effective than individual counseling, though few head-to-head comparisons have been conducted. Brief counseling with motivational interviewing (MI) strategies is also effective (42). A study of outpatients with serious mental illness randomized to receive a single 45-minute session of MI with personalized feedback versus interactive education found that the MI intervention significantly increased quit attempts by the 1-month follow-up [35% versus 14%; OR = 4.39 (1.44, 13.34)] (119). Although the quit attempts did not immediately translate to abstinence, quitting smoking often requires multiple attempts.

Integration of treatment into mental health care settings is likely to increase receipt of cessation services with added support from ongoing clinical monitoring. A large Veterans Affairs randomized trial across 10 sites with a total of 943 smokers with military-related PTSD compared integration of smoking cessation treatment within outpatient mental health care for PTSD versus referral to a smoking cessation clinic. The study found that integrated care was better than referral for prolonged abstinence [8.9% versus 4.5%; adjusted OR = 2.26 (1.30, 3.91)] (52).

The American Psychiatric Association (APA) (8) and the Royal College of Psychiatrists (100) have identified the psychiatric hospital setting as opportune for initiating cessation treatment. In a randomized trial with 224 patients recruited from a locked acute psychiatry unit with a 100% smoking ban, verified smoking 7-day point prevalence abstinence over 18-months follow-up was significantly higher for patients who received a computer-assisted cessation intervention with posthospitalization NRT (20.0%) versus usual care (7.7%) (95). A second study in a public psychiatric hospital with an ethnically diverse and low-income sample showed a similar pattern of effects (59). Among those with substance use disorders, a meta-analysis reported that counseling, contingency management, and relapse prevention improved continuous tobacco abstinence at 12 months (127).

#### Quitlines and web-based interventions

Tobacco quitlines (1-800-QUIT-NOW), developed in the 1990s, are an evidence-based treatment; every US state and territory currently has a quitline service that offers callers a combination of counseling and/or NRT (http://www.naquitline.org/). Tobacco quitlines provide cessation counseling with demonstrated efficacy and stronger effects in the general population when multiple counseling sessions are provided [RR = 1.37 (1.26, 1.50)] (42, 112, 118). A treatment modality with great public health potential, the national toll-free quitline number, created in 2004 (1-800-QUIT-NOW), provides cessation counseling to all Americans at no cost; yet, only 8% of smokers who are trying to quit and are aware of quitlines use them (110). Quitlines offer the benefits of convenience, anonymity, and low or no cost; these features can make quitlines especially useful to individuals with the most common psychiatric diagnosis (i.e., anxiety disorder).

Public health media campaigns combined with clinician education can help publicize and generate quitline referrals. In 2016, the Centers for Disease Control and Prevention’s Tips media campaign featured a smoker with depression, listing the quitline as a treatment resource (30). Prior to this campaign, the California quitline reported that nearly one in four of its callers met criteria for a current major depressive disorder; quit rates at 2-months follow-up were lower in this group (19%) than among callers without depression (28%). The low intensity and high accessibility of the treatment approach make it an important option for clinician referral, and supportive adjuncts may improve rates further (56). A 2016 randomized trial with 577 mental health patients in the Veterans Health Administration found that a specialized quitline for smokers with mental health concerns outperformed standard state quitlines, with significantly greater 30-day abstinence at 6 months (26% versus 18%) and greater patient satisfaction (106).

From a technology standpoint, there are a growing number of nontraditional modalities for cessation treatment. The National Cancer Institute (NCI) has four treatment web-sites (http://smokefree.gov, http://women.smokefree.gov, http://Espanol.smokefree.gov, http://teen.smokefree.gov) with resources for patients and providers, seven text-message programs, five smartphone apps, and multiple social media platforms. In 2013, more than 3 million smokers used the NCI websites, making the collection the most accessed smoking cessation web-site in the world (24). With the burst of available cessation programs online and via mobile applications, there is a need for greater evaluation, including among specialized groups (37).

#### Combination strategies

Clinical practice guidelines recommend combining medications with counseling to optimize quit rates (42), and evidence from network meta-analyses support the efficacy of combining short- and long-acting forms of NRT (25). Among smokers with substance use disorders, a meta-analysis found that cognitive behavioral therapy plus NRT improved quit rates at six months; in addition, a combination of bupropion, NRT, counseling, and contingency management improved quit rates at six months (127).

#### Provider training

The 2008 Public Health Service guidelines and the APA advocate treatment of tobacco use by psychiatrists and other mental health professionals (8, 42). MI and cognitive behavioral techniques are routinely used in mental health settings, and mental health providers have the skills to manage withdrawal symptoms and associated mood changes. Furthermore, tobacco can be easily integrated into provider assessments, treatment plans, and notes for prompting and follow-up. Brief advice to quit from a health care provider is associated with an increased likelihood of smoking cessation, and each visit to a mental health provider is a critical opportunity to reinforce the importance of quitting smoking (42).

Given the low rates of intervention and the high prevalence of tobacco use in those with mental illness with barriers to treating tobacco (1), training for psychiatrists and other mental health providers is essential yet still not widely implemented. In a survey of US psychiatry residency training programs, for example, only about half of training directors reported having a tobacco-cessation curriculum, and the time devoted to this topic was minimal (median = 1 h) (93). Recognition of this critical training gap led to development of Psychiatry RxforChange (http://rxforchange.ucsf.edu), a free online four-hour curricular resource for training mental health care professionals on treating tobacco use. The program has been associated with improvements in psychiatry residents’ knowledge, attitudes, confidence, and counseling behaviors for treating tobacco use among their patients; initial changes from pre- to post-training were sustained at three-month follow-up (92). The APA has developed a Tobacco Use Disorders work group to help disseminate resources and awareness of trainings such as Psychiatry RxforChange for mental health professionals.

#### Cost-effectiveness

Studies have long established that smoking cessation services are cost-effective (136). However, until recently, cost outcomes research in tobacco control has been limited to smokers without mental illness. Given that mental disorders topped the list of most costly conditions in 2013, with spending at $201 billion, treating tobacco use among those with mental illnesses is expected to have significant impacts on health care costs (4).

Three cost outcomes analyses have been published from randomized clinical trials of smokers with mental illness. In the above-mentioned veterans study with 943 smokers with PTSD (80), the mean cost of smoking cessation services was $1,286 in those randomized to integrated care (smoking cessation treatment within outpatient mental health care for PTSD) and $551 in those receiving standard care (referral to a smoking cessation clinic); the integrated care model added $836 in lifetime cost and generated 0.0259 quality-adjusted life years (QALYs), producing an incremental cost-effectiveness ratio of $32,257 per QALY, concluded to be 86% cost-effective (16). Efficiencies with computer-assisted cessation treatment and use of generic cessation pharmacotherapy can lower costs further. In a study with 322 smokers in outpatient treatment for clinical depression, intervention costs averaged $346 (including $221 for the computer intervention and $124 for brief counseling and pharmacotherapy), with 5.5% greater abstinence from smoking. If smoking cessation yields an additional 6 months of life (conservative estimate for smokers in general, not for those with mental illness), the cost would be $5,170 per life-year, which is cost-effective (17). With a similar intervention approach tested in inpatient psychiatry (95), the mean cost of smoking cessation services was $189 in the treatment group and $37 in the usual care condition. At 18 months, with more than twofold greater abstinence in the treatment group, the model projected that the intervention added $43 in lifetime cost and generated 0.101 additional QALYs, resulting in an incremental cost-effectiveness ratio of $428 per QALY, which was deemed highly cost-effective (18).

### Policy or Population Approaches

Policy-based approaches relevant for informing a comprehensive population-level tobacco control strategy include smoke-free laws, excise taxation on tobacco products, regulation of advertising and promotion, graphic warning labels, and plain packaging (132).

#### Smoke-free air

Home smoking bans reduce harmful secondhand smoke exposure, increase quit attempts and abstinence, and decrease cigarette consumption in adult smokers (82). A national US study found that statewide smoking bans in restaurants and bars were associated with reduced smoking among those with select psychiatric conditions (116). Furthermore, analysis of national data found that comprehensive smoking bans in the home and workplace were associated with a significantly reduced risk of developing major depression (14). Psychiatric hospitals are increasingly adopting smoking bans, although they are still not nationally mandated (71).

#### Tobacco taxes

In the United States, increasing tobacco taxes has produced the desired impact of both dissuading young people from starting to smoke and encouraging adult smokers to quit. When individuals have limited resources, at some point the costs of smoking (e.g., health harms, financial costs, social isolation) outweigh the perceived benefits or drive of the addiction. Analysis of data from the 2000–2001 Healthcare for Communities Survey demonstrated sensitivity to cigarette prices among individuals with alcohol, drug, or mental disorders: A 10% increase in cigarette prices was associated with 18% less smoking participation (84). The authors concluded that increasing cigarette taxes could be an effective strategy to reduce smoking in this group. Critical to pairing with the tax increases is the availability of cessation treatments via health care insurance coverage and resources such as the US quitline.

#### Health care coverage

In the United States, the Affordable Care Act (ACA) led to major changes to the health insurance market, which placed greater emphasis on prevention, including coverage for tobacco-cessation treatment. The ACA mandates comprehensive coverage for tobacco treatment for most private health plans and newly eligible Medicaid beneficiaries in states that expand Medicaid, including at least two tobacco-cessation attempts per year, four tobacco-cessation counseling sessions (each at least 10 minutes long), and any FDA-approved tobacco-cessation medication without cost-sharing or prior authorization for a 90-day treatment when prescribed by a health care provider. The ACA has the potential to dramatically improve access to clinical treatment of tobacco addiction by expanding benefits for traditionally medically underserved groups, including those with mental illness; in practice, however, not all insurers are advertising or implementing this benefit (68). The American Lung Association and addiction organizations are advocating for coverage of minimum benefits (6, 36). Moreover, the US Department of Health and Human Services issued specific guidance on insurance coverage of tobacco cessation as a preventive service under the ACA, clarifying that insurance plans should offer the benefits outlined above as part of standard health care (130). Concerning, however, is the ACA’s allowance for states to decide whether employers can charge smokers up to 50% more in premiums. Given the higher prevalence of smoking among those with less education and lower income, the unemployed, and those with mental illness, premium surcharges for smokers could dramatically raise the cost of health care for those least able to afford it.

Over 30 health care organizations have called for efforts to ensure that tobacco users in the United States are aware of and have barrier-free access to all evidence-based FDA-approved therapies and counseling, as mandated by the ACA and recommended by clinical practice guidelines. Incentives are in place for health care organizations to provide these services, given that tobacco-cessation treatments are cost-effective. For example, Massachusetts saved more than $3 for every $1 spent on cessation services for state Medicaid program beneficiaries (103). Investment in comprehensive tobacco cessation at the state and federal levels is warranted, as is continued research on novel medication development and delivery, diagnostics for precision medicine, and technological innovations in counseling engagement and reach.

## CONCLUSION

This review considers the complexity of factors that contribute to the high prevalence of tobacco use among individuals with psychiatric disorders. Because multiple levels of determinants are involved, as conceptualized in the HAVE framework, multilevel interventions are needed. Public health strategies include individual treatments with cessation support and medications directed at the host; regulation of tobacco products, including nicotine and flavorings targeting the agent as well as regulations on tobacco-industry marketing, advertising, and distribution that target the vector; and last, professional training and policies to create tobacco-free supportive environments with access to affordable care. A comprehensive effort is needed and warranted to address the significant harms of tobacco use, the leading preventable cause of death in the general population and in individuals with mental illness. These efforts are needed to achieve equity toward the 2025 Healthy People goal of reducing US adult tobacco use to 12% (134). Attention to all subgroups, including smokers with mental illness, is critical.

## SUMMARY POINTS

1. Tobacco use remains the leading preventable cause of death worldwide, disproportionately affecting individuals with mental illness and substance use disorders.
2. Cigarettes are the most common form of tobacco, though newer products such as electronic nicotine delivery systems (e.g., e-cigarettes, vape pens) are gaining in popularity and provide nicotine, the psychoactive and addictive component in tobacco.
3. Although there have been public health gains in tobacco control, the tobacco industry continues to propagate this major worldwide health burden, for example through research and product development and targeted advertising.
4. Mental health and substance use treatment providers have historically not addressed tobacco use disorders out of (false) concerns that doing so would compromise other outcomes.
5. There are effective and evidence-based methods to treat tobacco at the individual level, including seven FDA-approved cessation medications, quitlines, and counseling/groups; most efficacious are combinations of these treatments.
6. On a larger policy level, public health outcomes can change through product regulations, provider coverage of cessation treatments, and enactment of smoke-free air policies, especially in mental health and addiction treatment settings.
7. The sustained efforts of clinical providers, policy makers, and researchers are needed to address the major public health harms of tobacco use.
